# The Leukotriene Receptor Antagonist Montelukast as a Potential COVID-19 Therapeutic

**DOI:** 10.3389/fmolb.2020.610132

**Published:** 2020-12-17

**Authors:** Ludwig Aigner, Frank Pietrantonio, Diana Marisa Bessa de Sousa, Johanna Michael, Daniela Schuster, Herbert Anton Reitsamer, Horst Zerbe, Michael Studnicka

**Affiliations:** ^1^Institute of Molecular Regenerative Medicine, Paracelsus Medical University Salzburg, Salzburg, Austria; ^2^Spinal Cord Injury and Tissue Regeneration Center Salzburg (SCI-TReCS), Paracelsus Medical University Salzburg, Salzburg, Austria; ^3^Austrian Cluster for Tissue Regeneration, Vienna, Austria; ^4^IntelGenx Co5rp., Saint-Laurent, QC, Canada; ^5^Department of Pharmaceutical and Medicinal Chemistry, Institute of Pharmacy, Paracelsus Medical University Salzburg, Salzburg, Austria; ^6^Department of Ophthalmology and Optometry, University Clinic Salzburg, Paracelsus Medical University, Salzburg, Austria; ^7^Research Program of Experimental Ophthalmology and Glaucoma Research, Paracelsus Medical University Salzburg, Salzburg, Austria; ^8^Department of Pulmonary Medicine, University Clinic Salzburg, Paracelsus Medical University Salzburg, Salzburg, Austria

**Keywords:** leukotrienes, leukotriene receptor antagonist (LTRA), viral pneumonia, COVID-19, inflammation

## Abstract

The emergence and global impact of COVID-19 has focused the scientific and medical community on the pivotal influential role of respiratory viruses as causes of severe pneumonia, on the understanding of the underlying pathomechanisms, and on potential treatment for COVID-19. The latter concentrates on *four* different strategies: (i) antiviral treatments to limit the entry of the virus into the cell and its propagation, (ii) anti-inflammatory treatment to reduce the impact of COVID-19 associated inflammation and cytokine storm, (iii) treatment using cardiovascular medication to reduce COVID-19 associated thrombosis and vascular damage, and (iv) treatment to reduce the COVID-19 associated lung injury. Ideally, effective COVID-19 treatment should target as many of these mechanisms as possible arguing for the search of common denominators as potential drug targets. Leukotrienes and their receptors qualify as such targets: they are lipid mediators of inflammation and tissue damage and well-established targets in respiratory diseases like asthma. Besides their role in inflammation, they are involved in various other aspects of lung pathologies like vascular damage, thrombosis, and fibrotic response, in brain and retinal damages, and in cardiovascular disease. In consequence, leukotriene receptor antagonists might be potential candidates for COVID-19 therapeutics. This review summarizes the current knowledge on the potential involvement of leukotrienes in COVID-19, and the rational for the use of the leukotriene receptor antagonist montelukast as a COVID-19 therapeutic.

## Severe Acute Respiratory Syndrome Coronavirus 2 (Sars-Cov-*2)* and Coronavirus Disease-2019

Severe Acute Respiratory Syndrome Coronavirus 2 (SARS-CoV-2) is a novel enveloped RNA beta-coronavirus that emerged in December 2019 in Wuhan, Hubei Province, China, resulting in clusters of pneumonia outbreaks and human infections. In February 2020 the World Health Organization (WHO) announced the official name of the disease as “coronavirus disease-2019” (COVID-19). Coronaviruses are enveloped RNA viruses highly pathogenic to humans (Coleman and Frieman, 2014). In the past 20 years, *two* highly infectious coronaviruses gave rise to epidemics on a global scale i.e., severe acute respiratory syndrome coronavirus (SARS-CoV) (Ksiazek et al., 2003) and Middle East respiratory syndrome coronavirus (MERS-CoV) (de Groot et al., 2013). As of 11th Nov 2020, almost 52 million cases of COVID-19, including >1,200,000 deaths have been reported in over 190 countries (https://coronavirus.jhu.edu/map.html).

The incubation period for COVID-19 is estimated to be within 14 days following exposure, in most cases 4 to 5 days (Guan et al., 2020; Li et al., 2020). The clinical spectrum of COVID-19 syndrome ranges from asymptomatic infection to severe pneumonia, acute respiratory distress syndrome (ARDS), and death, based on 72,314 cases of COVID-19 from the Chinese Center for Disease Control and Prevention (Wu and McGoogan, 2020). In this study, 81% of cases were reported to be mild, 14% severe, and 5% were critical. In 1,482 hospitalized patients with laboratory-confirmed COVID-19 in the United States the most common presenting symptoms were cough (86%), fever or chills (85%), and shortness of breath (80%), diarrhea (27%), and nausea (24%) (Garg et al., 2020). Laboratory findings commonly reported in COVID-19 include leukopenia and lymphopenia, and elevations in aminotransferase levels, C-reactive protein, D-dimer, ferritin, and lactate dehydrogenase (Lovato A, 2020).

Patients with severe COVID-19 typically present with hypoxemia and need hospitalization. In adults with COVID-19 and acute hypoxemic respiratory failure, conventional oxygen therapy may be insufficient to meet the patient's oxygen demand. As such, further supportive options include high flow nasal cannula oxygen, non-invasive positive pressure ventilation, or invasive mechanical ventilation. In patients with COVID-19 from Wuhan, China, it was observed that over 20% of hospitalized patients with COVID-19 pneumonia required intensive care with respiratory support (Huang et al., 2020; Wang et al., 2020). Patients with COVID-19 pneumonia requiring ICU compared to non-ICU patients were older (median age = 66 years vs. 51 years) and more likely to be burdened with underlying co-morbid conditions (72 vs. 37%) (Wang et al., 2020).

## SARS-COV-2 Infection and Covid-19 Pathogenesis

**Figure 1:**
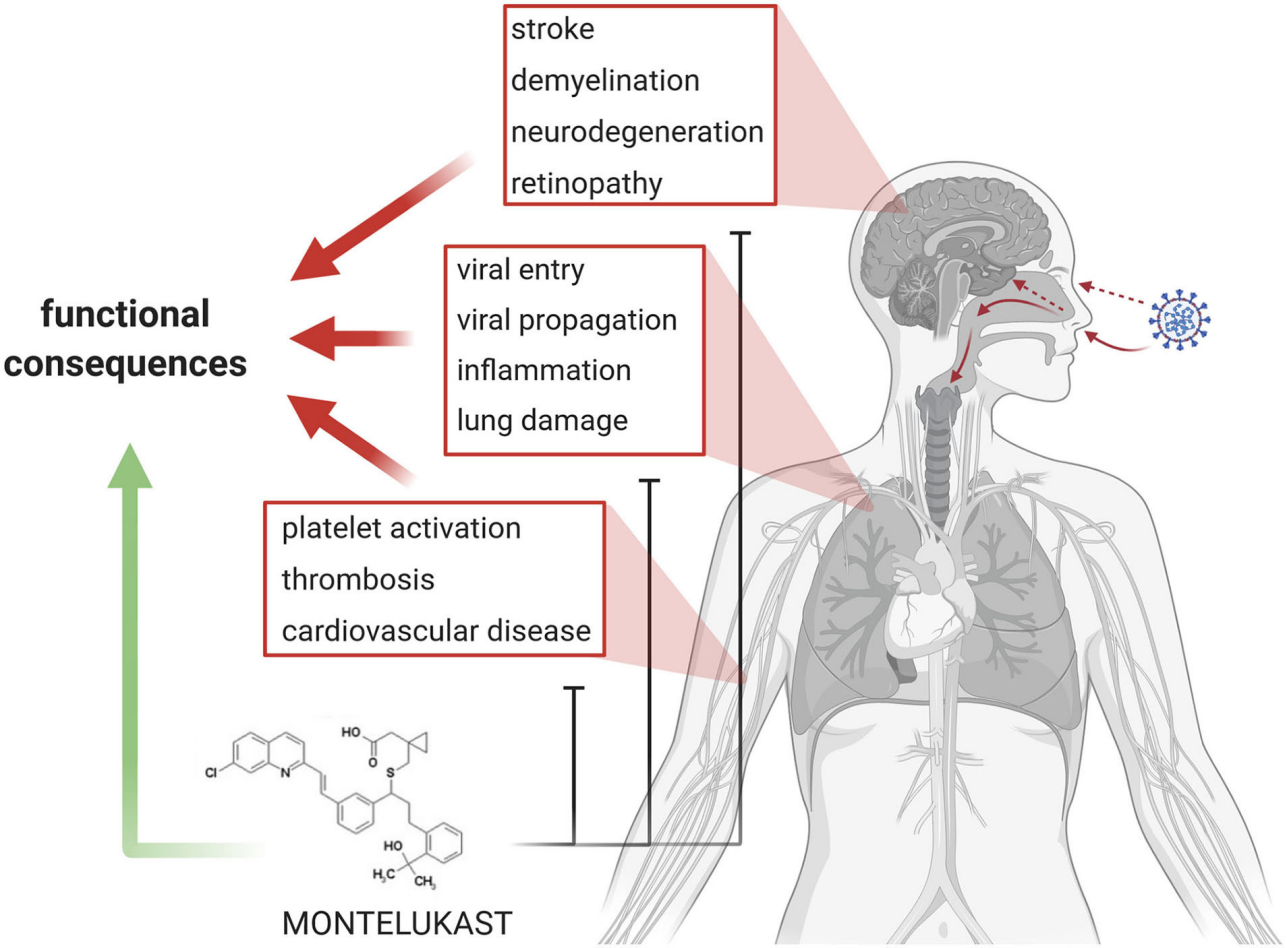
Summary and Concept. In the center of current COVID-19 research and therapy development are viral entry and propagation, inflammation, lung damage, as well as platelet activation/aggregation, thrombosis, and cardiovascular diseases. Emerging topics in COVID-19 are stroke, demyelination, and neurodegeneration. The various aspects lead to loss of organ function in the lung, brain and eye, and eventually heart. Montelukast might be effective in addressing the various detrimental processes and might promote functional recovery.

In brief, SARS- CoV-2 infection and COVID-19 pathogenesis can be summarized as follows: (i) entry of the virus into the upper and lower respiratory tract, cellular infection, replication, and propagation of the virus, (ii) inflammation, (iii) thrombosis and endothelial damage, and (iii) end organ damage (Figure 1). Finally, patients die because of respiratory failure due to interstitial pneumonia, cardiogenic shock or ARDS, while survivors recovering from COVID-19 often can develop fibrotic lung lesions.

### SARS-CoV-2 Infection and Propagation

Anti-viral treatment through inhibition of viral entry and/or inhibition of its propagation is certainly an attractive approach to reduce the risk of infection and to limit COVID-19 associated symptoms. At present, anti-viral medications that had originally been developed to treat HIV, Ebola, Hepatitis C, Influenza, SARS, or MERS virus infections are tested in COVID-19 patients. A fundamental understanding of the mechanisms of COVID-19 infection and propagation might open new avenues for the design of effective and specific treatments. Viral entry into target cells is facilitated through binding of the virus's spike (S) protein to the cellular receptor angiotensin-converting enzyme 2 (ACE2) as the entry receptor, similar to what has been described for SARS-CoV (Li et al., 2003; Zhou, P., et al., 2020). It further requires S protein cleavage/priming by the cellular protease Transmembrane Protease Serine 2 (TMPRSS2), which allows fusion of viral and cellular membranes. ACE2 and TMPRSS2 are expressed by a variety of cells of different organs, remarkably high expression is present in olfactory epithelium cells identifying the olfactory mucosa cells as one possible route of infection. In addition, ACE2 and TMPRSS2 expression is found in cells of the cornea of the eye and in the intestine. This suggests alternative routes of COVID-19 infections, for example through the eye, the tear ducts, or through the gastrointestinal tract. Viral propagation requires the main protease Mpro, also called 3CLpro, which is essential for cleaving the polypeptides that are translated from the viral RNA. Mpro is certainly a highly attractive drug target, however, the development of Mpro inhibitors has only recently started (Zhang et al., 2020).

### COVID-19 Associated Inflammatory Response

COVID-19 infections are associated with severe inflammation, and various anti-inflammatory treatments are currently under clinical investigations. Inflammation is partially triggered by virus induced cell cytolysis in the lung, which in turn results in enhanced production of inflammatory cytokines and chemokines by infected cells. Additional mechanisms include delayed induction of antiviral interferon response as a result of virus-escape mechanisms, such as the production of interferon inhibitory proteins (Channappanavar and Perlman, 2017; Merad and Martin, 2020).

Post-mortem analysis illustrates the excessive inflammatory response to SARS-CoV-2, which is thought to be the major contributor to disease severity and mortality in patients with COVID-19. This inflammatory response includes increased levels of inflammatory markers in the blood (including C-reactive protein, ferritin, and D-dimers), increased neutrophil/lymphocyte ratio and increased serum levels of several inflammatory cytokines, for example IL-1, IL-6, and TNF-α, and chemokines, as well as extensive lymphopenia and substantial infiltration of monocytes, macrophages and neutrophils in the lungs, heart, spleen, lymph nodes, and kidney (Huang et al., 2020; Mehta et al., 2020; Merad and Martin, 2020).

Dysregulated activation mechanisms of the mononuclear phagocyte compartment are likely contributors to COVID-19 associated hyper-inflammation (Schulert and Grom, 2015; Mehta et al., 2020). In various respiratory conditions including viral pneumonias levels of pro-inflammatory cytokines are elevated and various immune cells appear in lung tissue and contribute to symptoms such as fever and to tissue reactions such as fibrosis (Kritas et al., 2020). A similar situation appears in COVID-19, where levels of pro-inflammatory cytokines are elevated in the lung and in bronchial cells (Huang et al., 2020). In post-mortem lungs patients with coronavirus infections, there is extensive cellular infiltration by macrophages as well as accumulation of high levels of interferon-γ (IFNγ), IL-6, IL-12, transforming growth factor-β (TGFβ), CCL2, CXCL10, CXCL9, and IL-8 (Channappanavar and Perlman, 2017).

Severe COVID-19 patients typically experience a “cytokine storm syndrome” or now commonly coined as “cytokine storm,” characterized by a severe and sudden onset of a cytokine cascade or hypercytokinemia which in turn can result in ARDS, multi-organ failure, and death (Coperchini et al., 2020). The cytokine storm results from the cumulative effects of a combination of several immune-active molecules. The release of large quantities of interferons, interleukins, chemokines, colony-stimulating factors, and TNF-alpha, which represent the main components involved in the development of the cytokine storm, precipitate, and sustain the aberrant systemic inflammatory response. The “cytokine storm” is possibly the most dangerous and potentially life-threatening aspect related to COVID-19, and can result in ARDS, multi-organ failure, and death.

A common occurrence observed in many patients with severe COVID-19 is the presence of T cell lymphopenia, which is more pronounced in the CD8+ T cell compartment, although CD4+ T cell counts were also observed to be low. T cell counts were significantly reduced in COVID-19 patients, and were negatively correlated with increased serum levels of TNF, IL-6, and IL-10 (Diao et al., 2020). The surviving T cells appeared to be functionally exhausted (Diao et al., 2020). The potential mechanisms responsible for T cell depletion are still unclear; however, the hypothesis that depletion is related to direct infection of T cells by SARS-CoV-2 virus, at least in the case of patients with COVID-19, has to date not been substantiated.

### COVID-19 Patients Show Dysregulation of Hemostasis and Vascular Damage

Among patients, hospitalized for severe SARS-CoV-2 infection, COVID-19 is frequently associated with coagulation abnormalities (Al-Ani et al., 2020; Marietta et al., 2020). Several hematological and coagulation parameters are altered, including prolonged prothrombin and activated prothrombin times, enhanced D-dimer levels, as well as reduced platelet counts (Al-Ani et al., 2020; Becker, 2020; Terpos et al., 2020; Xu et al., 2020), with patients critically ill showing the most prominent alterations (Zhang, Y., et al., 2020). The hypercoagulative state observed in COVID-19 patients might be related to the strong pro-inflammatory response unleashed following viral infection (Marietta et al., 2020). Inflammatory mediators are known to promote platelet reactivity, activation of the coagulation system, and down-regulation of anticoagulant mechanisms favoring coagulation and thrombosis (Esmon, 2005). At the pulmonary level, cellular damage originating from viral infection, but also due to mechanical ventilation, may promote platelet activation and aggregation, leading to thrombus formation (Xu, X., et al., 2020; Zhang, Y., et al., 2020). Increased thrombogenesis may also contribute to thrombocytopenia observed in COVID-19 patients, a condition associated with poor prognosis and high mortality (Al-Ani et al., 2020; Liu et al., 2020). As highlighted in a recent position paper by the European Society of Cardiology (ESC) Working Group for Atherosclerosis and Vascular Biology, and the ESC Council of Basic Cardiovascular Science (Evans et al., 2020), SARS-CoV-2 virus infections affect the cardiovascular system with the clincial consequences of myocarditis, arrhythmias, and myocardial damage. Besides that, the vasculature is affected in COVID-19 patients, both directly by the SARS-CoV-2 virus, and indirectly as a result of the systemic inflammatory cytokine storm (Evans et al., 2020).

### COVID-19 Patients Develop Severe Acute and Chronic Lung Pathologies

On chest X-rays patients with COVID-19 show abnormalities that vary from patient to patient; nevertheless, patients typically demonstrate bilateral multi-focal opacities (Shi et al., 2020). CT chest scans also vary in terms of abnormalities; however, a common observation was peripheral ground-glass opacities with the development of areas of consolidation later in the clinical course. Also, imaging may be normal early after infection, and abnormalities may appear even in asymptomatic patients with COVID-19.

Histopathological examination of COVID-19 lung tissue shows cellular infiltrates indicating diffuse alveolar damage with cellular fibromyxoid-organizing exudates, accompanied by pneumocyte desquamation and hyaline membrane formation, indicating features usually seen in ARDS (Xu, X., et al., 2020). Also, pulmonary edema associated with hyaline membrane formation suggestive of early-phase ARDS is typically evident. Multinucleated syncytial cells, atypical enlarged pneumocytes characterized by large nuclei, amphophilic granular cytoplasm, and prominent nucleoli are observed in the intra-alveolar spaces, indicating changes seen in viral infections.

In addition, COVID-19 patients show extensive mucus secretion in both lungs, and signs of pulmonary interstitial fibrosis are typically evident in post-mortem COVID-19 lungs (Jain, 2020). The severity of pulmonary complications in COVID-19 is closely linked to IL-6 peak levels (Russell et al., 2020). The over-activation of mast cells and release of cytokines might also have a crucial role in the development of pulmonary fibrosis in COVID-19, particularly in populations pre-disposed to develop diseases related to mast cell activation (Theoharides, 2020). Chronic lung diseases like pulmonary fibrosis may develop in COVID-19 patients who recovered (Wang J., et al., 2020).

## Leukotrienes In Lung Pathologies and Covid-19

COVID-19 associated inflammatory responses involve the participation of the innate and the adaptive immune system as well as its associated cellular players and components. Besides the inflammatory overload, thrombosis, vascular damage, and fibrotic response are typical features of COVID-19 pathology. In the search for a common denominator involved in the modulation of these various aspects of COVID-19 pathology we identified leukotrienes (LTs), in particular the cysteinyl-leukotrienes (Cys-LTs) and their receptors as potential drug targets. Indeed, very recent literature hypothetically argues for the use of LT receptor antagonists in the treatment of COVID-19 patients (Almerie and Kerrigan, 2020; Fidan and Aydogdu, 2020), and a first Phase III randomized controlled double-blind clinical trial testing the Cys-LT receptor antagonist montelukast in COVID-19 patients has been announced (https://clinicaltrials.gov/ct2/show/NCT04389411). The objective of COSMO (COvid-19 Symptom MOntelukast) trial is to determine the efficacy of montelukast in reducing the severity of COVID-19 symptoms. The primary objective is to test the efficacy of the standard and approved dose of 10 mg/day of montelukast compared to placebo in reducing the risk of acute care visits and hospital admissions for COVID-19 patients.

The next paragraphs elaborate on Cys-LTs and their role in lung pathologies with a special focus on COVID-19 related pathogenesis and summarize the rational and the current state of preclinical and clinical development of montelukast as a potential therapeutic for the treatment of COVID-19 patients.

### Leukotrienes and Leukotriene Receptors

LTs are eicosanoids and inflammatory mediators produced by various cell types including leukocytes. One subclass, i.e., cysteinyl-leukotrienes (CysLTs) are well-known in respiratory medicine as they trigger bronchoconstriction in asthma and cause inflammation in asthma and allergic rhinitis (Peters-Golden and Henderson, 2007; Okunishi and Peters-Golden, 2011). CysLTs bind to specific CysLT receptors (CysLTRs) namely, CysLTR1, CysLTR2, P2Y12, GPR99, and GPR-17. These G protein-coupled receptors are expressed on the outer membrane of a variety of cells including immune and inflammatory cells (i.e., basophils, mast cells, dendritic cells, eosinophils, monocytes/macrophages, B cells, CD4^+^ T cells, and to a lesser degree on neutrophils and CD8^+^ cells), endothelial cells and platelets (Peters-Golden and Henderson, 2007; Okunishi and Peters-Golden, 2011). CysLTR1 is mostly expressed in lymphoid cells of the spleen and peripheral blood leukocytes (Lynch et al., 1999) and also at lower levels in lung, colon, small intestines, kidney, liver, heart, pancreas, and brain (Nonaka et al., 2005). CysLTR2 is expressed in spleen, heart, peripheral blood leukocytes, and lung (Takasaki et al., 2000) and moderately expressed in the central nervous system with higher expression levels within the spinal cord and pituitary (Nothacker et al., 2000). P2Y12 is mainly expressed by platelets but also in various other cell types including cells of the upper and lower respiratory tract, where it mediates LT-induced effects such as eosinophilic inflammation in asthma (Foster et al., 2013; Suh et al., 2016). GPR99 is a high-affinity receptor for LTE4 and involved in vascular damage, mucin production and mucosal swelling (Bankova et al., 2016) [for review see Yokomizo et al. (2018)]. GPR-17 has been described in various stem and progenitor cells, and other somatic cells. It has affinity to two families of ligands i.e., nucleotide sugars nucleotide sugars (UDP, UDP-galactose, and UDP-glucose) and Cys-LTs (LTD4, LTC4, and LTE4) (Ciana et al., 2006). In the context of respiratory diseases, GPR17 might be involved in modulating pulmonary immune-related inflammations (Zhan et al., 2018). Overall, besides the clear involvement of CysLTR1 as a mediator of eicosanoids in inflammation, the precise role of other leukotriene receptors in inflammation is still under discussion. For example, the oxoglutarate receptor GPR99 may be activated by CysLTs in mice and *in vitro*, but its role in human CysLT-induced effects remains to be established (Back et al., 2014).

### Cysteinyl Leukotrienes in Lung and Respiratory Disease Pathologies

LTs play a pivotal role in the acute phase of respiratory conditions such as (i) asthma, (ii) viral pneumonia, (iii) acute lung injury (ALI), (iv) systemic inflammatory response syndrome (SIRS), (v) ARDS, and (vi) pulmonary fibrosis (Beller et al., 2004; Caironi et al., 2005; Horiguchi et al., 2007; Okunishi and Peters-Golden, 2011; Al-Amran et al., 2013). LTs mediate various molecular and cellular pathologies in respiratory disease, and LT inhibition alleviates respiratory pathology (Sorkness, 1997; McMillan, 2001; Scott and Peters-Golden, 2013). Whether LTs are involved in SARS-CoV-2 induced pneumonia and COVID-19 pathology is unclear at present. Nevertheless, the similarities of symptoms such as cough and fever, dyspnoea, pneumonia, respiratory failure, and sepsis between COVID-19 associated and not-associated respiratory conditions, and the similarities in the various aspects of respiratory disease pathology such as inflammation, thrombosis and vascular damage, and fibrotic reactions strongly argue for a role of LTs in SARS-CoV-2 associated lung diseases.

The vast amount of knowledge on the role of CysLTs in lung disease has been generated in the field of asthma, where CysLTs mediate inflammation, induce bronchoconstriction, increase microvascular permeability, and increase mucus production (Peters-Golden, 2008). CysLTRs have been identified as therapeutic targets, and CysLTR antagonists such as montelukast are in clinical use in asthma for more than two decades. The fact that CysLTs are involved in the various aspects of respiratory disease pathologies such as inflammation, thrombosis and vascular damage, and fibrotic remodeling provides a rationale for inhibition of LTs and the use of montelukast in respiratory diseases beyond asthma, for example in viral pneumonia related to SARS-CoV-2 infections.

Besides the well-established role of CysLTs, leukotriene B4 and ist receptors might also be relevant targets in the context of inflammation related to asthma (Ro et al., 2019). For example, leukotriene B4 (LTB_4_) is present at higher concentrations in sputum of patients with severe asthma compared to those with mild asthma. Moreover, LTB_4_ receptors are involved in the pathogenesis of neutrophil-dominant pulmonary inflammation in an animal model. Nevertheless, due to the fact that the role of CysLTs in the pathogenesis of asthma, in particular in the inflammation that is related to asthma, is far more established compared to the one of LTB_4_, the present review focuses primarily on CysLTs and their receptors as putative targets in lung diseases including COVID-19. Moreover, as the need of a COVID-19 therapeutic is more than timely, we are focusing on compounds are already approved medications and ready to be repurposed. Again, while Cys-LTR antagonists are approved medications, LTB_4_ antagonists are available for preclinical studies but not as approved medications.

## Montelukast as a Potential Covid-19 Therapeutic

Montelukast was developed as a highly selective CysLTR1 antagonist, which is currently used and approved for the treatment of asthma, allergic rhinitis, exercise-induced bronchoconstriction, and acetylsalicylic acid-sensitive asthmatic patients. Its primary mode of action is via prevented signaling of LTs by blockage of CysLTR1. This prevents the production of reactive oxygen species (ROS) and LTB4 and inhibits inflammatory cytokine production through blocking the MAPK-p38 and NF-kB pathways (Anderson et al., 2009), which has been demonstrated in TNF-alpha-stimulated IL-8 expression in U937 cells (Tahan et al., 2008), in human monocytic leukemia cell line (Maeba et al., 2005), and in peripheral blood derived macrophages (Lin et al., 2018). In addition to the inhibition of CysLTR1, montelukast also antagonizes the GPR17 LT receptor (Ciana et al., 2006).

### Effects of Montelukast in Viral Infection and Propagation

In 2017, Cardani et al. described an animal model of Influenza A pneumonia demonstrating a role of alveolar macrophages in preventing lethality (Cardani et al., 2017). In this study, the preventive effect of the alveolar macrophages was causally linked to a down-regulation of the LT pathway. Furthermore, genetic and pharmacologic inhibition of LT synthesis as well as blockade of CysLTRs using the LTR antagonist zafirlukast reduced susceptibility to Influenza A infection and protected these mice from lethal infections (Cardani et al., 2017). Based on the similar molecular pharmacology montelukast might have a similar anti-viral activity as zafirlukast. This, however, requires experimental proof. Nevertheless, the anti-viral activity of montelukast has been demonstrated in for the Zika virus, a RNA virus similar to Sars-CoV-2. Here, montelukast disrupted the integrity of the virions to release the viral genomic RNA and thus irreversibly inhibited viral infectivity (Chen et al., 2019).

Propagation of the SARS-CoV-2 virus requires the main protease Mpro, which is processing and cleaving the viral polypeptides (Zhang et al., 2020). A study by Wu et al. generated computational 3D homology models for 19 viral targets and used molecular docking to predict drugs that could bind to the respective binding sites. Montelukast was predicted to bind Mpro with high affinity to its catalytic site of Mpro, presenting montelukast as a potential Mpro inhibitor (Wu et al., 2020). This may directly modulate and inhibit viral replication in COVID-19. However, this computational prediction has still to be experimentally tested.

### Montelukast to Reduce Inflammation in Lung and Respiratory Diseases

In human asthma, the anti-inflammatory effects of montelukast in asthma are well-established [for review see Diamant et al. (2009), Okunishi and Peters-Golden (2011), Paggiaro and Bacci (2011)]. For example, in adults with asthma montelukast treatment reduced serum CRP, decreased serum and sputum eosinophil counts, levels of eosinophil cationic protein and of IL-8; montelukast decreased sputum levels of myeloperoxidase, and increased serum and sputum levels of the anti-inflammatory cytokine IL-10 (Kanniess et al., 2002; Stelmach et al., 2005; Allayee et al., 2007). Also, in asthmatic children montelukast reduced the levels of exhaled nitric oxide (Straub et al., 2005). In mycoplasma pneumonia, montelukast decreased serum MCP-1, PCT, ICAM-1, CXCL8, CRP, IFN-γ, and IL-17 levels and peripheral blood Th1 and Th17 numbers, while it increased serum IL-4 and TGF-β levels and peripheral blood Treg and Th2 content (Wu et al., 2019).

In a number of animal models of respiratory diseases montelukast demonstrated anti-inflammatory activities. For example, in an animal model of respiratory syncytial virus (RSV) induced bronchiolitis montelukast prevented airway hyper-responsiveness and inflammation (Han et al., 2010). Additionally, Wedde-Beer et al. (2002) reported that treatment with montelukast suppresses vascular permeability of airway mucosa in a rat model of RSV infection. In two studies on haemorrhagic shock induced lung-injury montelukast reduced IL-6 and TNF-α levels (Horiguchi et al., 2007), alleviated lung injury and decreased serum levels of lung myositis associated antibodies, as well as bronchoalveolar lavage fluid LTB(4), LTC(4), and total protein (Al-Amran et al., 2013). Also, montelukast attenuated LPS-induced lung inflammation in a model of acute respiratory distress syndrome (Davino-Chiovatto et al., 2019). On the cellular level montelukast suppressed the release of pro-inflammatory mediators such as IL-8 and RANTES in nasal airway epithelial cells *in vitro* (Scaife et al., 2013).

### Montelukast Affects Platelets and Alleviates Vascular Damages

Increasing evidence demonstrates platelet involvement in different lung diseases, such as asthma (Kowal et al., 2006). In asthmatic individuals, airway inflammation is associated with intravascular platelet activation (Sullivan et al., 2000), with platelets contributing to the activation and infiltration of eosinophils and T cells to the bronchial wall (Sullivan et al., 2000; Kowal et al., 2006; Benton et al., 2010; Trinh et al., 2019). Interestingly, combination of clopidogrel, an antiplatelet drug, and montelukast in the treatment of asthma seems to alleviate airway inflammation in animal experiments (Trinh et al., 2019). As previously discussed, platelet aggregation and thrombosis are two important events triggered by SARS-CoV-2 infection. It is, thus, expectable that COVID-19 patients have elevated levels of activated platelets, which might contribute to the host response to the virus. Besides being involved in recruitment and transmigration of immune cells to inflamed tissue (Leiter and Walker, 2019), activated platelets also release multiple inflammatory molecules, which support the activation and recruitment of immune cells, increased of vascular permeability and a pro-inflammatory environment (Leiter and Walker, 2019).

Interestingly, in the context of allergen induced airway inflammation, platelet function seems to be influenced by CysLTs (Liu et al., 2015). Platelets express CysLT1R and CysLT2R (Hasegawa et al., 2010), and platelet activation by LTC4 has been shown in mouse platelets (Cummings et al., 2013). LTC4 platelet stimulation led to upregulation of plasma membrane P-selectin expression, a molecule involved in leukocyte recruitment and release of inflammatory mediators (i.e., thromboxane A2, CXCL4, and RANTES) (Cummings et al., 2013). Furthermore, *in vitro* stimulation of human platelets with LTC4, D4, and E4 also induced the release of RANTES (Hasegawa et al., 2010), a chemokine involved in the recruitment and migration of leukocytes to inflammatory sites (Marques et al., 2013). CysLTs-mediated platelet release of RANTES could be partial abrogated by Pranlukast, a CYSLT1R antagonist (Hasegawa et al., 2010). These observations suggest that CysLTs might be involved in platelet activation and that the use of CysLT1R antagonists such as montelukast might be advantageous in the treatment of inflammatory states, particularly in combination with antiplatelet drugs, such as clopidogrel (Foster et al., 2013; Suh et al., 2016).

### Montelukast and the Potential to Limit Acute and Chronic Lung Tissue Damage in COVID-19

Currently, there are no data yet available demonstrating that montelukast prevents lung damage in COVID-19 patients. Also, while montelukast inhibits pulmonary inflammatory circuits in the asthmatic lung, its effects in COPD is less established. Nevertheless, montelukast reduced the levels of procollagen type I carboxy-terminal propeptide (PICP-1), a marker for collagen-synthesis and airway remodeling in hypertonic saline solution–induced sputum in children with asthma (Tenero et al., 2016). In animal models of lung injury, montelukast has been shown to ameliorate tissue damage. For example, montelukast reduced sepsis-induced lung and renal injury in rats, a model of systemic inflammatory response syndrome (Khodir et al., 2014). In a model of hemorrhagic shock induced lung injury, montelukast reduced the total lung injury score (Al-Amran et al., 2013).

## Montelukast in Covid-19—A Therapy Beyond Lung?

There is increasing evidence that COVID-19 pathology is not limited to the lung but also affects other organs, in particular brain and eye. A retrospective study from Mao et al. of 214 hospitalized COVID-19 patients revealed that 36.4% of patients displayed neurologic symptoms, like dizziness, headache and impaired consciousness (Mao et al., 2020). Also gustatory and olfactory dysfunctions have been reported (Wang, L., et al., 2020). Severe neurologic complications, like encephalitis, demyelination and stroke have also been described in association with COVID-19 in rare cases (Poyiadji et al., 2020) [reviewed in (Asadi-Pooya and Simani, 2020; Montalvan et al., 2020; Zanin et al., 2020)]. These neurological symptoms point toward a potential of Sars-CoV-2 to damage the central nervous system (CNS). Also, ophthalmological changes such as conjunctivitis, conjunctival hyperemia, increased secretion, chemosis, and epiphora have been described in patients with COVID-19 (Wu, P., et al., 2020). In addition, OCT based scans of the retina show evidence for pathologies in the ganglion cell layer and the inner plexiform layer and the inner plexiform layer in COVID-19 patients (Marinho et al., 2020). Additional observations using color fundus photography and red-free imaging showed cotton wool spots and microhaemorrhages along the arcade vessels at the macular border indicating ischemia, blockage of axoplasmatic transport in the ganglion cells as well as breakdown of the blood retina barrier (Marinho et al., 2020). Cases of temporal mild and severe vision loss binocular and monocular have been reported, and non-arteritic posterior ischemic optic neuropathies were discussed as possible reasons for acute vision loss (Selvaraj et al., 2020).

Direct entry of the Sars-CoV-2 into the brain and eye has been postulated. A possible direct entry way of Sars-CoV-2 into the brain is via the lamina cribrosa of the ethmoid bone, which was shown for Sars-CoV infections (Netland et al., 2008). Sars-CoV-2 might also enter the brain by infecting endothelial cells in the cerebral circulation (Baig et al., 2020). The receptor ACE2 is expressed on cells of the central nervous tissue, namely neurons and glial cells (Harmer et al., 2002) making them potential target cells of Sars-CoV-2 in the CNS (Baig et al., 2020). It is known that other members of the coronavirus family are able to enter the CNS and that an infection can lead to symptoms of multiple sclerosis and encephalitis (Bohmwald et al., 2018; Natoli et al., 2020). Presence of Sars-CoV-2 in the CNS has already been documented (Zhou et al., 2020), which demonstrates a neuroinvasive potential of this virus. It was suggested, that a neuroinvasion of Sars-CoV-2 could be involved in respiratory failure in severe COVID-19 patients (Li, Y. C., et al., 2020), which of course needs further research.

There is controversy about the ocular surfaces being an entry and production site for the Sars-CoV-2 Virus. At least in patients with PCR positive tear fluid samples spread of the virus from the nasopharynx through the nasolacrimal duct onto the ocular need to be considered as a possible route for viral entry onto the ocular surface. However, recent evidence suggests that ACE-2 and TMPRSS2 are located on the conjunctival epithelium of adult humans. This makes most of the outer ocular surface susceptible to infections with Sars-CoV-2 (Collin et al., 2020). In patients with Sars-CoV-2 positive tear fluid on the ocular surface, the eye is a possible site of transmission of the virus. Taking measurements of intraocular pressure with air-puff-tonometry was discussed as a possible mechanism transmitting the virus via aerosol propagation (Lai et al., 2020). Although no infection with Sars-CoV-2 via this pathway was reported, as a precaution, most societies have warned using this technique in COVID-19 patients. Cauterization of infected conjunctiva also created aerosols and might as well be a possible mechanism for virus transmission.

Neurological and visual impairments due to Sars-CoV-2 infections might directly and/or indirectly damage the CNS including brain and retina, which could potentially have long-term consequences. Naughton et al. has raised concerns that COVID-19 might contribute to further cognitive decline in patients with Alzheimer Disease (Naughton et al., 2020). Given the relative short time that Sars-CoV-2 is present we can by now only speculate on possible mid- and long-term consequences for COVID-19 survivors. Given the potential of Sars-CoV-2 to infect cells of the CNS concerns arise that an infection might facilitate and fasten cognitive decline in patients with mild cognitive impairment or dementia. These effects could either be due to direct neuropathogenic capacity of Sars-Cov-2 in the brain or an indirect consequence of the systemic inflammation.

A huge body of preclinical experiments has demonstrated that montelukast promotes CNS repair, regeneration, and rejuvenation in animal models of aging, chronic neurodegenerative disease and acute CNS lesions. For example, in aged rats, montelukast re-activated neurogenesis, reduced neuroinflammation, restored the blood-brain-barrier, and improved learning and memory (Marschallinger et al., 2015). Montelukast alleviated damage, restored fiber connectivity, and improved neurological function in an animal model of acute stroke (Gelosa et al., 2019). It reduced seizures and restored blood-brain-barrier function in an animal model of epilepsy (Lenz et al., 2014), and reduced the alpha-synuclein load and restored memory in an animal model of Lewy-Body dementias (Marschallinger et al., 2020), In an animal model of multiple sclerosis, montelukast reduced T-cell chemotaxis and restored integrity of the blood-brain-barrier (Wang et al., 2011).

Although very speculative, it can be expected that Sars-CoV-2 infections damage the brain involving various cellular levels and mechanisms including blood-brain-barrier, neuroinflammation, neurons and glia, as well as progenitor cells. Montelukast has been shown to act in a protective and regenerative mode of action at these various cells and compartments, and might therefore have a potential to protect and repair from Sars-CoV-2 induced brain damages. The same might account for retinal damages, as montelukast has been shown to prevent from retinal capillary degeneration in an animal model of early diabetic (Bapputty et al., 2019).

Increasing awareness exists on the role of endothelial cells as a prime target for SARS-CoV-2 infections and on cardiovascular symptoms in COVID-19 patients (Evans et al., 2020). Again, here, montelukast might be protective and alleviate cardiovascular symptoms. For example, it has been demonstrated that montelukast suppressed the expression of adhesion molecule such as VCAM-1 and E-selectin and reduces monocyte adhesion to human umbilical vein endothelial cells *in vitro* (Di et al., 2017). Importantly, a nationwide cohort study in sweden demonstrated that montelukast intake was associated with a reduced risk for recurrent cardiovascular diseases (Ingelsson et al., 2012). Moreover, we recently demonstrated that montelukast reduced brain damage in ischemic rodents and promoted structural and functional recovery after stroke (Gelosa et al., 2019). Therefore, in summary, montelukast might exert a number of extra-pulmonary protective effects in the context of COVID-19.

## Monteluakst for Covid-19 Patients—A Drug Delivery Case?

As suggested in this review, there is a strong scientific rational for repurposing of montelukast as a COVD-19 therapeutic. Currently, montelukast is marketed under the brand name Singulair® and in several generic products in oral tablet forms. In adult patients, the recommended daily approved dose is a 10-mg tablet. These tablet forms present a number of limitations such as inconsistent solubility, uptake, and bioavailability. Although montelukast is freely soluble in water, its solubility is markedly and significantly increased above pH 7.5 and drastically reduced under acidic conditions normally found in the gastrointestinal tract, in particular in the stomach (Okumu et al., 2008). This explains why absorption of montelukast into the blood stream is relatively slow and inconsistent with maximum concentrations occurring between 2–4 h following consumption. Thereby, montelukast use is limited to chronic conditions rather than for rapid acute treatment. Indeed, a major obstacle limiting the absorption of montelukast is presented by its insufficient solubility and the rate of dissolution from the tablet form. Uptake and bioavailability of montelukast is further determined by pharmacogenetics [for review see (Thompson et al., 2016)]. For example, more than 20% of the population is not responding to montelukast with a clinical benefit (Noonan et al., 1998). Among the various genetic reasons are variations in the *SLCO2B1* gene coding for the organic anion transporting *OATP2B1*, which has been associated with altered absorption of montelukast (Mougey et al., 2009). Uptake of montelukast was further modifed by the intake of citrus juice (Mougey et al., 2011).

Besides the physico-chemical and genetic basis for the insufficient uptake and bioavailability of montelukast in its current tablet form, a further drawback is the inadequateness of montelukast tablets for patients suffering from dysphagia such as elderly patients. Most importantly, for patients that are intubated or require ventilation, an oral montelukast tablet wouldn't be the application of choice. As the most critical COVID-19 patients are elderly people, and in severe cases require intubation and ventilation, alternative routes of application might be favorable.

One alternative drug delivery mode, which would be highly suitable for elderly patients, and in particular for patients with intubation or ventilation, are mucoadhesive buccal films. Indeed, we have developed a buccal mucoadhesive film formulation of montelukast, which in a recent Phase I study showed safe and tolerable in healthy subjects, and provides a reduction in first-pass-effect and a 52% higher bioavailability compared to the regular montelukast tablet. Given the potential mechanism of action of montelukast in COVID-19 and the added benefit of the buccal film application, the latter might be a meaningful and effective therapeutic in patients with COVID-19.

## Recent Concerns on Safety of Montelukast

As of 2017, there were over 30 million prescriptions of montelukast in the United States (https://clincalc.com/DrugStats/Drugs/Montelukast). The clinical efficacy of montelukast in the approved indications has been well-established in randomized clinical trials and the safety has been proven in numerous years of use on the global market (Schoors et al., 1995; Noonan et al., 1998; Storms et al., 2001). Despite the vast clinical potential of montelukast and other leukotriene modifying compounds in various diseases, there is concern about the adverse drug reactions associated with montelukast treatment in children as well as in adults. Besides reports of allergic granulomatous angiitis, some neuropsychiatric symptoms were observed with montelukast use. Among those, depressions, aggressions, headaches and nightmares occur in a certain percentage of children and adults, and even cases of suicide after use of montelukast have been reported (Haarman et al., 2017). Importantly, the FDA recently re-evaluated the benefits and risks of montelukast (Singulair and generics) use, strengthened existing warnings about serious behavior and mood-related changes with montelukast and determined that a *Boxed Warning* was appropriate. The underlying mechanisms of such side effects are completely unknown. In consequence to this remaining risk of adverse events, and as there are currently no predictive biomarkers available for such adverse events, COVID-19 patients would require thorough examinations for such adverse events while they are on the drug.

## Summary and Conclusion—Repurposing Montelukast for Covid-19 Treatment

To date, there are no marketed and effective antiviral drug products or biologics available for the control of SARS-CoV-2, other than symptomatic clinical treatment strategies for COVID-19. LTs might be involved in COVID-19 pathology. The LT receptor antagonist montelukast might provide antiviral activity through modulation of the Mpro inhibitor site and as such may inhibit viral replication. Montelukast is an anti-inflammatory drug and might alleviate vascular and parenchymal damage. Several investigations in China and Italy looking at comorbidities or pre-existing medical conditions in laboratory-confirmed COVID-19 patients did not find asthma among the comorbidities; similarly, asthma was not reported when patients died as a result of SARS-CoV-2 infection. The use of asthma medication like montelukast might have had a role in minimizing the clinical presentation of this comorbidity. Given that to date, there are minimal to no effective strategies in the armamentarium against this debilitating and lethal COVID-19 disease, this treatment modality should be considered. Montelukast might not only alleviate pathology but promote structural and functional recovery (for summary see Figure 1).

## Author Contributions

LA wrote major parts of the manuscript. FP, DB, JM, DS, HR, HZ, and MS wrote specific chapters. LA and MS conceived the study. All authors read and approved the final manuscript.

## Conflict of Interest

FP and HZ are affiliated to Intelgenx, a company who has commercial interest in Montelukast. LA is scientific advisor for Intelgenx. JM has a Ph.d., fellowship from Intelgenx. The remaining authors declare that the research was conducted in the absence of any commercial or financial relationships that could be construed as a potential conflict of interest.

## Abbreviations

ACE 2: angiotensin-converting enzyme
ALI: acute lung injury
ARDS: acute respiratory distress syndrome
CCL2: CC-chemokine ligand 2
CNS: central nervous system
COSMO: COvid-19 Symptom MOntelukast
Covid19: corona virus disease 2019
CRP: c reactive protein
Cys-LTs: cysteinyl-leukotrienes
CysLTR1: Cysteinyl-leukotriene receptor 1
CysLTR2: Cysteinyl-leukotriene receptor 2
CXCL8: CXC motif chemokine ligand 8
CXCL9: CXC motif chemokine ligand 9
CXCL10: CXC motif chemokine ligand 10
FDA: Food and Drug administration
GPR99: G-protein coupled receptor 99
GPR-17: G-protein coupled receptor 17
HIV: human immunodeficiency virus
ICAM-1: intercellular adhesion molecule 1
ICU: intensive care unit
IFNγ: interferon gamma
IL-1: interleukin 1
IL-6: interleukin 6
IL-8: interleukin 8
IL-12: interleukin 12
IL-17: interleukin 17
LT: leukotrienes
LTC4: leukotriene C4
LTD4: leukotriene D4
LTE4: leukotriene E4
MAPK: mitogen-activated protein kinase
MCP-1: monocyte chemotactic protein 1
MTK: Montelukast
Mpro: main protease
MERS-CoV: middle eastern respiratory syndrome corona virus
PCT: procalcitonin
PICP-1: procollagen type I carboxy-terminal propeptide
RANTES: regulated on activation, normal T cell expressed and secreted
ROS: reactive oxygen species
Sars-Cov 2: severe acute respiratory syndrome corona virus 2
TMPRSS2: Transmembrane Protease Serine 2
TNF-α: tumor necrosis factor alpha
TGFβ: transforming growth factor-β
WHO: world health organization.
